# Loss of CARM1 is linked to reduced HuR function in replicative senescence

**DOI:** 10.1186/1471-2199-14-15

**Published:** 2013-07-09

**Authors:** Lijun Pang, Haiyan Tian, Na Chang, Jie Yi, Lixiang Xue, Bin Jiang, Myriam Gorospe, Xiaowei Zhang, Wengong Wang

**Affiliations:** 1 Department of Biochemistry and Molecular Biology, Peking University health Science Center, 38 Xueyuan Road, Beijing 100191, P. R. China; 2 Laboratory of Molecular Biology and Immunology, National Institute on Aging, National Institutes of health, 251 Bayview Blvd., Baltimore, MD 21224, USA

**Keywords:** CARM1, HuR methylation, mRNA turnover, Replicative senescence

## Abstract

**Background:**

The co-activator-associated arginine methyltransferase 1 (CARM1) catalyzes the methylation of HuR. However, the functional impact of this modification is not fully understood. Here, we investigated the influence of HuR methylation by CARM1 upon the turnover of HuR target mRNAs encoding senescence-regulatory proteins.

**Results:**

Changing the methylation status of HuR in HeLa cells by either silencing CARM1 or mutating the major methylation site (R217K) greatly diminished the effect of HuR in regulating the turnover of mRNAs encoding cyclin A, cyclin B1, c-fos, SIRT1, and p16. Although knockdown of CARM1 or HuR individually influenced the expression of cyclin A, cyclin B1, c-fos, SIRT1, and p16, joint knockdown of both CARM1 and HuR did not show further effect. Methylation by CARM1 enhanced the association of HuR with the 3′UTR of p16 mRNA, but not with the 3′UTR of cyclin A, cyclin B1, c-fos, or SIRT1 mRNAs. In senescent human diploid fibroblasts (HDFs), reduced CARM1 was accompanied by reduced HuR methylation. In addition, knockdown of CARM1 or mutation of the major methylation site of HuR in HDF markedly impaired the ability of HuR to regulate the expression of cyclin A, cyclin B1, c-fos, SIRT1, and p16 as well to maintain a proliferative phenotype.

**Conclusion:**

CARM1 represses replicative senescence by methylating HuR and thereby enhancing HuR’s ability to regulate the turnover of cyclin A, cyclin B1, c-fos, SIRT1, and p16 mRNAs.

## Background

RNA binding protein HuR, the ubiquitously expressed member of Hu RNA binding proteins [1], is functionally involved in the regulation of mRNA stabilization, translation, and export [2-4]. Among the cellular events that are influenced by HuR and its target mRNAs is the process of replicative senescence. Several studies have implicated HuR in regulating the turnover of cyclin A, cyclin B1, c-fos, SIRT1, and p16 mRNAs, as well as the nuclear export of HuR mRNA during replicative senescence [4-8].

Although the molecular events controlling HuR function are not fully understood, the cytoplasmic presence and post-translational modification (e.g., phosphorylation and methylation) of HuR are particularly important [2,9-11]. For example, the cell cycle checkpoint kinase Chk2 has been shown to interact with HuR and phosphorylate HuR at residues S88, S100, and T118; phosphrylation at S100 seems to be important for the dissociation of the HuR-SIRT1 mRNA complex in response to oxidative stress [6]. The cyclin-dependent kinase 1 (CDK1) phosphorylates the HuR hinge region at serine 202 and leads to enhanced HuR association with 14-3-3, thereby retaining HuR in the nucleus [11]. Additionally, studies by Doller and coworkers identified HuR as a substrate for both PKCα and PKCδ. The phosphorylation of HuR at S221 by both PKCα and PKCδ is also linked to HuR shuttling as well as the stabilization of COX-2 mRNA [12]. These findings underscore functional links between HuR and the aforementioned signaling cascades, which regulate HuR shuttling or its binding to target mRNAs.

Besides phosphorylation, methylation is another important post-translational modification of HuR. The co-activator associated arginine methyltransferase 1 (CARM1) catalyses the methylation of HuR and HuD *in vitro* and *in vivo*[13,14]. CARM1 has been reported to methylate the histones (e.g.,H3,H4) and transcriptional factors [e.g., p53,hormone-activated nuclear receptor (NR), etc.] at arginine residues, thereby activating gene transcription [15-18]. Methylation by CARM1 enhances the function of HuD and HuR in stabilizing TNF-α and SIRT1 mRNAs [14,19], respectively. The R217 present within the HuR hinge region is identified as the major methylation site by CARM1 [13]. It was proposed that the interaction of HuR nuclear ligands SETα/β, pp32, and APRIL with the HuR hinge region regulates HuR shuttling [2]. However, thus far, the functional impact of CARM1-mediated HuR methylation on replicative senescence and the precise mechanism underlying remain largely unexplored.

In this study, by using two approaches to mimic the hypomethylation of HuR, knockdown of CARM1 and mutation of HuR at the major methylation site (R217), we have investigated the functional impact of the CARM1-mediated HuR methylation and the mechanisms underlying in replicative senescence. Our results indicate that the methylation by CARM1 is critical for HuR to regulate the turnover of mRNAs encoding cyclin A, cyclin B1, c-fos, SIRT1, and p16 in replicative senescence.

## Results

### Methylation by CARM1 enhances the effect of HuR in regulating the turnover of cyclin A, cyclin B1, c-fos, SIRT1, and p16 mRNAs

**Figure 1:**
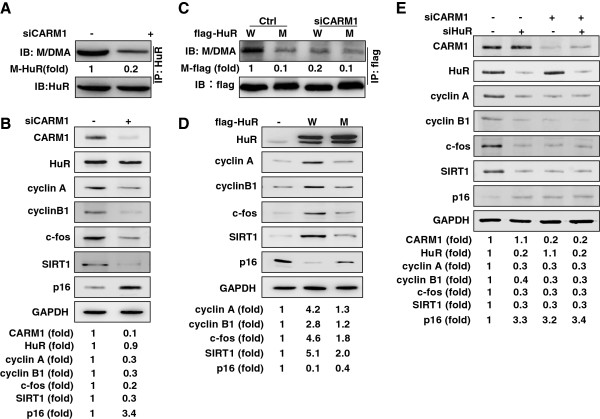
**Methylation by CARM1 enhances the regulating of cyclin A, cyclin B1, c-fos, SIRT1, and p16 by HuR. (A)** Forty-eighth after transfection of HeLa cells with CARM1siRNA (+) or a control siRNA (−), lysates were prepared for IP assays by using HuR antibody. The presence of total and methylated HuR in the IP materials was determined by Western blot analysis by using M/DMA and HuR antibodies, respectively. **(B)** Cell lysates described in Figure 1A were subjected to Western blot analysis to assess the protein levels of CARM1, HuR, cyclin A, cyclin B1, c-fos, SIRT1, p16, and GAPDH. Western blotting signals were quantified by densitometry. **(C)** HeLa cells were transfected with a vector expressing flag-HuR or flag-HuRΔ. Twenty fourh later, cells were further transfected with CARM1 siRNA or a control siRNA and cultured for an additional 48 h. Whole-cell lysates were prepared and subjected to IP assays by using anti-flag antibody (M2). Western blot analysis was performed to assess the total and methylation levels of flag-tagged HuR in the IP materials using M/MDA and flag antibodies, respectively. **(D)** HeLa cells were transfected with a vector expressing flag-HuR or flag-HuRΔ. Forty eighth later, lysates were prepared to assess the protein levels of CARM1, HuR, cyclin A, cyclin B1, c-fos, SIRT1, p16, and GAPDH by Western blot analysis, the signals of Western blotting were quantified by densitometry. **(E)** HeLa cells were either transfected with HuR or CARM1 siRNA or co-transfected with both siRNAs. Forty eighth later, Western blot analysis was performed to evaluate the levels of CARM1, HuR, cyclin A, cyclin B1, c-fos, SIRT1, p16, and GAPDH; Western blotting signals were quantified by densitometry. Data are representatives from 3 independent experiments.

Because HuR has been reported to stabilize cyclin A, cyclin B1, c-fos, and SIRT1 mRNAs, and destabilize p16 mRNA [5-7], we asked if CARM1-mediated HuR methylation influences the expression of these genes. To begin to answer this question, whole-cell lysates from Hela cells silenced CARM1 were subjected to immunoprecipitation assays (IP) by using HuR antibody, whereupon the levels of methylated and total HuR in the IP materials were determined by Western blot analysis using M/DMA and HuR antibodies, respectively. As shown in Figure 1A, the level of methylated HuR (M- HuR) was reduced by ~80% in cells with silenced CARM1, while total HuR levels remained unchanged. By Western blot analysis, knockdown of CARM1 reduced the protein levels of CARM1 (by ~90%), cyclin A (by ~70%), cyclin B1 (by ~70%), c-fos (by ~80%), and SIRT1 (by ~70%), and increased the protein level of p16 (by ~3.4 fold) (Figure 1B). In agreement with the results shown in Figure 1A, knockdown of CARM1 did not alter HuR protein abundance. These results suggest that CARM1-mediated methylation may enhance the effect of HuR in regulating the turnover of its target mRNAs. To confirm this point, the levels of cyclin A, cyclin B1, c-fos, SIRT1, and p16 in HeLa cells expressing flag-HuR (flag-HuR, W) or flag-HuRΔ [flag-HuRΔ, mutant (M)] were assessed by Western blotting. A mutant HuR bearing an arginine-to-lysine mutation on residue 217, identified as the major methylation site of HuR [13] was expressed as a fusion protein (flag-HuRΔ). After IP using anti-flag antibody (M2), the methylation status of flag-HuR and flag-HuRΔ was assessed by Western blotting using M/DMA antibody, as described in Figure 1A. As shown in Figure 1C, the methylation of flag-HuRΔ (M-flag) was reduced by ~80% relative to that of flag-HuR (lane 1 vs. lane 2). On the other hand, knockdown of CARM1 reduced the methylation status of flag-HuR (by ~90%) (lane 1 vs. lane 3), but not that of flag-HuRΔ (lane 2 vs. lane 4). We next tested the protein levels of cyclin A, cyclin B1, c-fos, SIRT1, and p16 in HeLa cells expressing flag-HuR (W) or flag-HuRΔ (M) by Western blotting. In keeping with previous findings [5-7], expression of flag-HuR (lanes 2, W) increased the levels of cyclin A (by ~4.2 fold), cyclin B1 (by ~2.8 fold), c-fos (by ~4.6 fold), and SIRT1 (by ~5.1 fold), and reduced the levels of p16 (by ~90%), compared to the levels of these proteins in cells transfected with the empty vector (lanes 1, -) (Figure 1D). However, expression of flag-HuRΔ (lanes 3, M) elicited a much weaker effect on protein levels: cyclin A increased only by ~1.3 fold, cyclin B1 by ~1.2 fold, c-fos by ~1.8 fold, and SIRT1 by ~2.0 fold, while p16 levels were reduced by ~60% (Figure 1D).

We next asked if CARM1 functions through methylating HuR. HeLa cells were transfected with HuR siRNA, CARM1 siRNA or both siRNAs, and 48 h later, the levels of cyclin A, cyclin B1, c-fos, SIRT1, and p16 were assessed by Western blot analysis. As anticipated, knockdown of HuR (lanes 2) or CARM1 (lanes 3) individually reduced the levels of cyclin A, cyclin B1, c-fos, and SIRT1, and increased the levels of p16 (Figure 1E). However, joint knockdown of HuR and CARM1 (lanes 4) was not more effective than individual knockdown of HuR or CARM1 (Figure 1E). In sum, by methylating HuR, CARM1 is able to regulate the expression of cyclin A, cyclin B1, c-fos, SIRT1, and p16.

**Figure 2:**
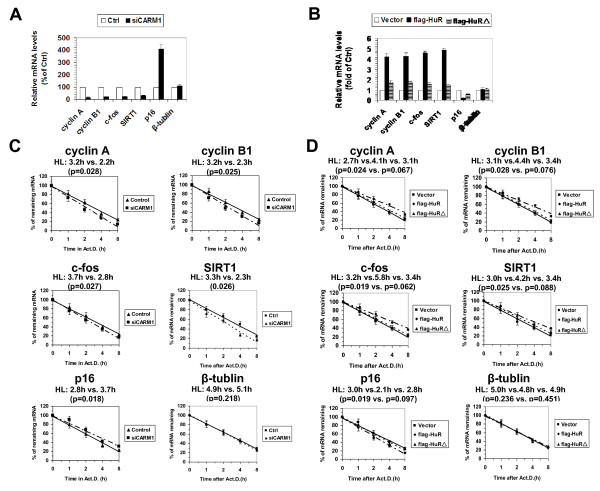
**Methylation by CARM1 influences the effect of HuR in regulating mRNA turnover. (A and B)** RNA was prepared from cells described in Figure 1A **(A)** and 1D **(B)** to assess the mRNA levels of cyclin A, cyclin B1, c-fos, SIRT1, p16, and β-tublin by real-time qPCR against GAPDH. **(C and D)** Cells described in Figure 1A and 1D were exposed to actinomycin D (2 μg/ml), whereupon the cellular RNA was isolated at times indicated. Real-time qPCR against GAPDH was performed to assess the half-lives of cyclin A, cyclin B1, c-fos, SIRT1, p16, and β-tublin mRNA, as described in Methods. The real-time qPCR data are represented as means ± SEM from 3 independent experiments. The statistic significance was analyzed by Student’s t test.

Next, we measured the levels and half-lives of cyclin A, cyclin B1, c-fos, SIRT1, and p16 mRNAs in cells described in Figure 1B and 1D, as described in ‘Methods’. As shown in Figure 2A, silencing CARM1 reduced the levels of cyclin A (by ~83%), cyclin B1 (by ~78%), c-fos (by ~76%), and SIRT1 (by ~69%), but induced that of p16 (by ~4.1 fold). As anticipated, knockdown of CARM1 shortened the half-lives of cyclin A (3.2 h vs. 2.2 h, p = 0.028), cyclin B1 (3.2 h vs. 2.3 h, p = 0.025), c-fos (3.7 h vs. 2.8 h, p = 0.027), and SIRT1 (3.3 h vs. 2.3 h, p = 0.026) mRNAs, and extended the half-life of p16 mRNA (2.8 h vs. 3.7 h, p = 0.018) (Figure 2C). As a negative control, knockdown of CARM1 did not influence the levels (Figure 2A) orhalf-lives (Figure 2C) of β-tublin mRNA (4.9 h vs. 5.1 h, p = 0.218). In keeping with previous findings [5-7], expression of flag-HuR (flag-HuR) increased the mRNA levels of cyclin A (by ~4.2 fold), cyclin B1 (by ~4.3 fold), c-fos (by ~4.6 fold), and SIRT1 (by ~4.9 fold), but decreased that of p16 (by ~76% ) (Figure 2B). The marked changes in steady-state levels for these mRNAs were due in part to changes in their stabilities [cyclin A mRNA (2.7 h vs. 4.1 h, p = 0.024), cyclin B1 mRNA (3.1 h vs. 4.4 h, p = 0.028), c-fos mRNA (3.2 h vs. 5.8 h, p = 0.019), SIRT1 mRNA (3.0 h vs. 4.2 h, p = 0.025) mRNAs, and p16 mRNA (3.0 h vs. 2.1 h, p = 0.019) (Figure 2D)]. In contrast, expression of flag-HuRΔ (flag-HuRΔ) had a much weaker effect, increasing cyclin A mRNA levels by ~1.7 fold, cyclin B1 mRNA by ~ 1.8 fold, c-fos mRNA by ~ 1.6 fold, and SIRT1 mRNA by ~ 1.5 fold, and reducing p16 mRNA by ~40% (Figure 2B). As above, these changes were due in part by changes in the half-lives of these mRNAs: cyclin A mRNA (2.7 h vs. 3.1 h, p = 0.067), cyclin B1 mRNA (3.1 h vs. 3.4 h, p = 0.076), c-fos mRNA (3.2 h vs. 3.4 h, p = 0.062), SIRT1 mRNA (3.0 h vs. 3.4 h, p = 0.088), and p16 mRNA (3.0 h vs. 2.8 h, p = 0.097) (Figure 2D)]. As a negative control, the levels (Figure 2B) and half-lives (Figure 2D) of β-tublin mRNA in cells expressing flag-HuR were comparable to that observed in cells expressing flag-HuRΔ (Figure 2B and 2D). These results suggest that methylation by CARM1 enhances HuR’s ability to regulate the turnover of cyclin A, cyclin B1, c-fos, SIRT1, and p16 mRNAs.

### Methylation by CARM1 enhances the association of HuR with p16 3′UTR, but not cyclin A, cyclin B1, c-fos, and SIRT1 3′UTR

**Figure 3:**
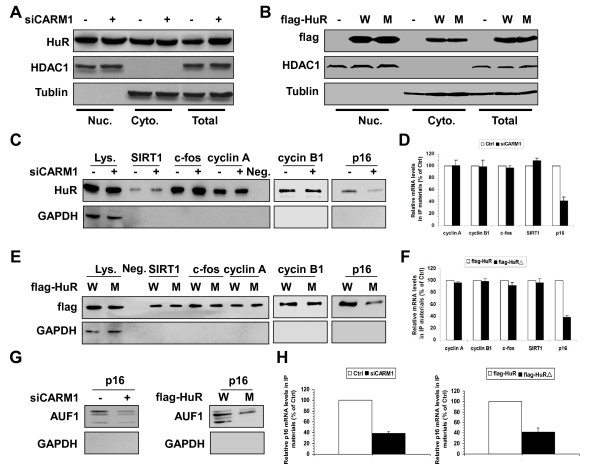
**Influences of CARM1-mediated methylation on the subcellular distribution and RNA-binding affinity of HuR. (A and B)** Western blot analysis was performed to assess the presence of endogenous HuR **(A)** as well as flag-tagged HuR (flag-HuR and flag-HuRΔ) **(B)** in whole-cell (Total, 10 μg), cytoplasmic (Cyto., 40 μg), and nuclear fractions (Nuc., 5 μg) prepared from cells described in Figure 1A and 1D. Assessment of the levels of cytoplasmic-specific tubulin and nuclear-specific HDAC1 served to verify the quality and equal loading of the cytoplasmic and nuclear preparations, respectively. **(C)** Cytoplasmic extracts (100 μg) described in Figure 3A were subjected to RNA pull-down assays using biotinylated 3′UTR fragments of cyclin A, cyclin B1, c-fos, SIRT1, and p16 to detect bound endogenous HuR by Western blotting. A 10-μg aliquot of whole-cell lysates (Lys.), binding of HuR and GAPDH to the beads (Neg.), and binding of GAPDH to the cyclin A, cyclin B1, c-fos, SIRT1, and p16 3′UTR were also tested. **(D)** Cytoplasmic extracts (100 μg) described in Figure 3A were subjected to RNP IP assays using anti- HuR antibody. The presence of cyclin A, cyclin B1, c-fos, SIRT1, and p16 mRNAs in the IP materials were assessed by real-time qPCR. **(E, F)** Cytoplasmic extracts (100 μg) described in Figure 3B were either subjected to RNA pull-down assays **(E)** or RNP IP assays **(F)** to assess the association of flag-HuR and flag-HuRΔ with the mRNAs of cyclin A, cyclin B1, c-fos, SIRT1, and p16, as described in Figure 3C and 3D. **(G, H)** Cytoplasmic extracts described in Figure 3A and 3B were either used for RNA pull-down assays **(G)** or used for RNP IP assays **(H)** to assess the association of AUF1 with p16 mRNA, as described in Figure 3C and 3D.

Since the changes in HuR target mRNA half-lives occurred in the cytoplasm, it is important to ask if methylation of HuR by CARM1 influenced the levels of cytoplasmic HuR. To this end, the cytoplasmic, nuclear, and whole-cell fractions from cells described in Figure 1B and 1D were prepared as described [9] and the presence of HuR in different cellular fractions was assessed by Western blot analysis. As shown in Figure 3A, knockdown of CARM1had no influence on the presence of HuR in the cytoplasm or the nucleus. Similarly, the total (lanes 8 and 9), cytoplasmic (lanes 2 and 3), and nuclear levels (lanes 5 and 6) of flag-HuR (W) and flag-HuRΔ (M) were relatively similar (Figure 3B). Therefore, methylation by CARM1 did not markedly alter the subcellular distribution of HuR.

Besides its localization, the interaction of HuR with target mRNAs influences its ability to regulate mRNA turnover or translation. To further test the influence of CARM1-mediated methylation on the interaction of HuR with RNA, biotinylated fragments of cyclin A, cyclin B1, c-fos, SIRT1, and p16 3′UTRs and cytoplasmic extracts described in Figure 3A and 3B were used for pull-down and RNP IP assays, as previously described [7]. As shown in Figure 3C and 3D, knockdown of CARM1 had no influence on the association of HuR with the 3′UTRs of cyclin A, cyclin B1, c-fos, and SIRT1 mRNAs. In addition, the association of flag-HuR (W) and flag-HuRΔ (M) with the 3′UTR of cyclin A, cyclin B1, c-fos, and SIRT1 was comparable (Figure 3E and 3F). However, changing HuR methylation either by silencing CARM1 (Figure 3C and 3D) or by mutating the HuR methylation site (Figure 3E and 3F) markedly lowered the association of HuR with p16 3′UTR. In a previous study, HuR and AUF1 were found to bind with the p16 3′UTR and destabilize p16 mRNA cooperatively [7]. To address whether methylation of HuR by CARM1 influences the association of AUF1 with p16 3′UTR, the lysates described in Figure 3C and 3E were used for pull-down and RNP IP assays. As shown in Figure 3G and 3H (left), in cells with silenced CARM1, the association of AUF1 with p16 3′UTR was markedly reduced. Moreover, in cells expressing flag-HuRΔ (M), the association of AUF1 with the p16 3′UTR was substantially weaker than that observed from cells expressing flag-HuR (W) (Figure 3G and 3H, right). These results suggest that methylation by CARM1 enhances the association of HuR with the p16 3′UTR, and in turn, enhances the association of AUF1 with the p16 3′UTR.

### CARM1- HuR regulatory process impacts on replicative senescence

**Figure 4:**
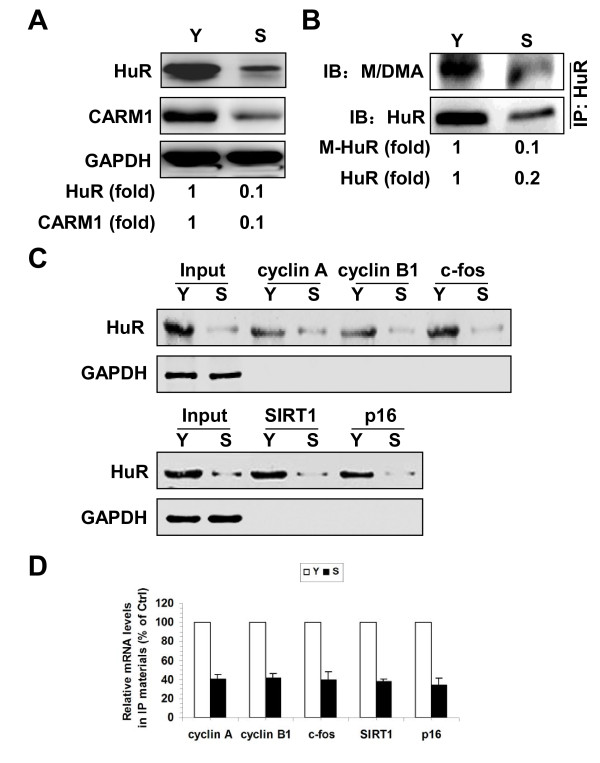
**CARM1 and methylated HuR reduce replicative senescence. (A)** Western blot analysis of CARM1, HuR, and GAPDH protein levels in early-passage (Young, ~27 pdl, Y) and late-passage (Senescent, ~60 pdl, S) 2BS cells. **(B)** HuR was immunoprecipitated from the whole cell lysates (100 μg) described in Figure 3A, whereupon the total or methylated HuR was assessed by Western blot analysis using HuR or M/DMA antibodies, respectively. Western blotting signals were quantified by densitometry. Data are representative from 3 independent experiments. **(C)** Cytoplasmic extracts prepared from cells described in Figure 4A were subjected to RNA pull-down assays using biotinylated 3′UTR fragments of cyclin A, cyclin B1, c-fos, SIRT1, and p16 to detect bound HuR by Western blotting. A 10-μg aliquot of whole-cell lysates (Input) and binding of GAPDH to the cyclin A, cyclin B1, c-fos, SIRT1, and p16 3′UTR were also tested. **(D)** Cytoplasmic extracts described in Figure 4C were subjected to RNP IP assays using anti-flag antibody, the presence of cyclin A, cyclin B1, c-fos, SIRT1, and p16 mRNAs in the IP materials were assessed by real-time qPCR.

HuR regulates replicative senescence at least in part by stabilizing cyclin A, cyclin B1, c-fos, and SIRT1 mRNAs as well as destabilizing p16 mRNA [5-7]. To address whether the methylation of HuR by CARM1 impacts upon replicative senescence, we first examined the levels of CARM1 and the methylation status of HuR in early-passage [Young, ~27 population doublings (pdl)] and late-passage [Senescent, ~60 (pdl)] human diploid fibroblasts (2BS) by Western blotting. As shown in Figure 4A, the levels of CARM1 in senescent 2BS cells (S) were markedly reduced relative to the levels in young 2BS cells (Y). As a positive control, HuR protein levels in the senescent cells were also potently reduced, in keeping with previous findings [4]. To evaluate the methylation status of HuR in senescent cells, HuR was immunoprecipitated from lysates prepared from cells described in Figure 4A. The IP materials then were subjected to Western blot analysis using the M/DMA antibody. As anticipated, the levels of both methylated HuR (M- HuR) and total- HuR ( HuR) were markedly reduced in senescent cells (Figure 4B). In addition, reduction of HuR and CARM1 protein levels as well as the HuR methylation levels was accompanied with the decrease of the association of HuR with cyclin A, cyclin B1, c-fos, SIRT1, and p16 mRNAs in senescent cells (Figure 4C and 4D).

**Figure 5:**
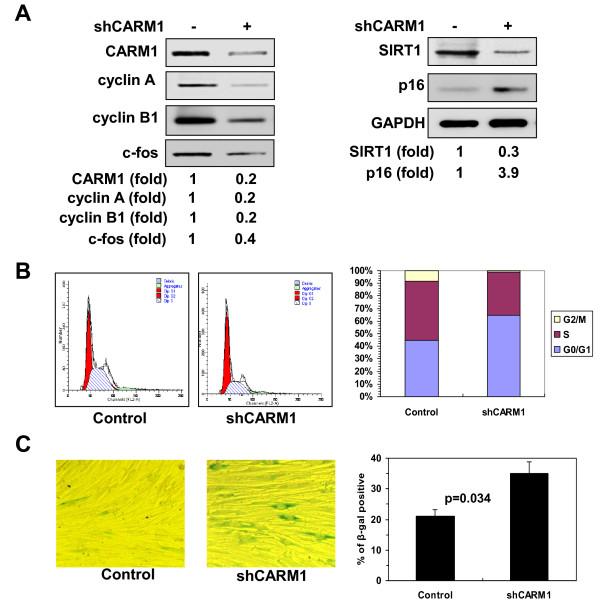
**Knockdown of CARM1 reduces the levels of HuR target mRNAs and accelerates cell senescence. (A)** Human diploid fibroblasts (2BS) were stably transfected with a vector expressing CARM1 shRNA or control shRNA. The levels of CARM1, cyclin A, cyclin B1, c-fos, SIRT1, p16, and GAPDH were assessed by Western blotting and the signals quantified by densitometry. **(B, C)** Cells described in Figure 5A were subjected to FACS analysis **(B)** and SA-β-gal staining **(C)** to assess the cell cycle distribution and senescent status. The SA-β-gal staining was represented as means ± SDs from three independent experiments. The statistic significance was analyzed by Student’s t test.

**Figure 6:**
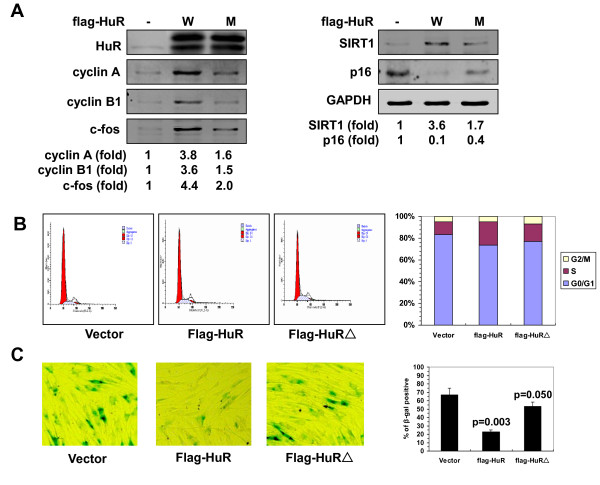
**Mutation of the methylation site attenuates the effect of HuR in regulating mRNA turnover and cell senescence. (A)** Human diploid fibroblasts were stably transfected with a vector expressing flag-HuR (W) or flag-HuRΔ (M), or an empty vector (−). Western blot analysis was performed to assess the protein levels of HuR, cyclin A, cyclin B1, c-fos, SIRT1, p16, and GAPDH, and quantified by densitometry. **(B,C)** Cells described in Figure 6A were subjected to FACS analysis **(B)** and SA-β-gal staining **(C)** to analyze the cell cycle distribution and cell senescent status, as described in Figure 5B and 5C. Values of the SA-β-gal staining represent means ± SDs of the results from three independent experiments. Statistical significance was analyzed by Student’s t test.

Next, we evaluated the functional impact of the methylation of HuR by CARM1 in replicative senescence. 2BS cells were transfected with a vector expressing CARM1 shRNA or control shRNA and selected by G418 for 3 weeks. As indicated in Figure 5A, knockdown of CARM1 led to a reduction in the levels of cyclin A (by ~80%), cyclin B1 (by ~80%), c-fos (by ~60%), and SIRT1 (by ~70%), and to an induction in p16 levels (by ~3.9 fold) (Figure 5A). Knockdown of CARM1 decreased S and G2 compartments (Figure 5B) and increased the number of SA-β-gal positive cells (21% vs. 35%, p = 0.034) (Figure 5C). In accordance with previous findings [5-7], 2BS cells stably transfected with a vector expressing flag-HuR (W) increased the protein levels of cyclin A (by ~3.8 fold), cyclin B1 (by ~3.6 fold), c-fos (by ~4.4 fold), and SIRT1 (by ~3.6 fold), and decreased the levels of p16 (by ~90%) (Figure 6A), thereby increasing the S and G2 compartments (Figure 6B), and decreasing the number of SA-β-gal positive cells (67% vs. 23%, p = 0.003) (Figure 6C). In contrast, expressing flag-HuRΔ (M) was less effective than expressing flag-HuR (W) in inducing the levels of cyclin A (by ~1.6 fold), cyclin B1 (by ~1.5 fold), c-fos (by ~2.0 fold), and SIRT1 (by ~1.7 fold), and reducing p16 protein level (by ~60%) (Figure 6A). Accordingly, expressing flag-HuRΔ was less effective than expressing flag-HuR in increasing the S and G2 compartments (Figure 6B), as well as in decreasing the number of SA-β-gal positive cells (67% vs. 53%, p = 0.050) (Figure 6C). These findings suggest that the CARM1- HuR regulatory process impacts upon the process of replicative senescence.

## Discussion

The present study provides novel insight into the regulation of HuR function in replicative senescence. By modulating the HuR methylation status, we gained evidence to support the view that methylation by CARM1 critically affects HuR’s ability to regulate the levels of cyclin A, cyclin B1, c-fos, SIRT1, and p16 as well as the process of cell senescence (Figures 1, 2, 4, 5, and 6).

Reduced HuR levels and cytoplasmic concentration have been linked to the lower expression of cyclin A, cyclin B1, c-fos, and SIRT1 and the increased expression of p16 in replicative senescence [5-7]. Thus, signaling events that regulate the relative levels of HuR in the nucleus and the cytoplasm may also involve in the process of cell aging [4]. Although the mechanism controlling HuR shuttling has not been fully elucidated, the hinge region of HuR is of great importance for its nuclear localization [2]. Because the Arg217 localizes at the hinge region, we hypothesized that the methylation of HuR by CARM1 may elevate the expression of HuR targets by increasing the cytoplasmic presence of HuR. However, lowering HuR methylation by either silencing CARM1 or mutating the major methylation site had no effect on the shuttling of HuR (Figure 3A, B). It is also possible that methylation of HuR affects its RNA-binding affinity because methylation may affect the interaction of HuR with the hinge-binding proteins, which could in turn modulate RNA binding [13]. Indeed, ithas been reported that methylation by CARM1 could enhance the association of HuR with SIRT1 3′UTR during the differentiation of human embryonic stem cells (hESC) [19]. Here, loss of HuR methylation either by silencing CARM1 or by mutating the major methylation site did not influence the association of HuR with the 3′UTRs of SIRT1, cyclin A, cyclin B1 or c-fos. However, methylation of HuR by CARM1 enhanced the association of HuR to p16 3′UTR (Figure 3C-H). Therefore, whether methylation by CARM1 influences the binding affinity of HuR may not only depend on the methylation status of HuR and the mRNAs targeting by HuR, but also on the type and physiologic state of the cell in which the HuR-mRNA interaction occurs.

In addition to HuR and HuD, methylation of poly(A)-binding protein 1, CA150, the SAP49, SmB, and the U1 small nuclear RNP-specific protein U1C has been implicated in the process of mRNA stabilization as well as the 5′ splice site selection of the pre-mRNA splicing [20-22]. Methylation of hnRNP A2, the nuclear poly (A)-binding protein PABPN1, and the poly (U)-binding proteins Sam68 and SLM by PRMT1 influences their function and proper localization [23-25]. Because both the expression and methylation status of HuR significantly decline with replicative senescence (Figure 4), it is challenging to evaluate the contribution of CARM1-mediated methylation to the reduction of HuR methylation in senescent cells. However, the evidence presented here (Figures 5 and 6) suggests that the CARM1- HuR regulatory process do contribute to the regulation of genes associated with replicative senescence. Although the links between human aging and replicative senescence are not fully understood, senescent cells accumulate with advancing age and actively contribute to physiologic and pathologic changes of aging. Our finding that CARM1 contributes to the alterations of cyclin A, cyclin B1, c-fos, SIRT1, and p16 in replicative senescence warrants a careful look at CARM1 in other models of aging. In light of the fact that HuR is also an important regulator of cell death [26,27], cell differentiation [19,28,29], and human cancer [10,30-32], we postulate that CARM1-HuR regulatory process may impact upon these processes as well.

## Conclusions

CARM1-mediated protein methylation enhances the function of HuR upon the turnover of mRNAs encoding cyclin A, cyclin B1, c-fos, SIRT1, and p16. Methylation by CARM1 did not influence HuR subcellular distribution or association with the 3′UTRs of cyclin A, cyclin B1, c-fos, and SIRT1 mRNAs, but it enhanced the association of HuR with the p16 3′UTR. By methylating HuR, CARM1 critically regulates replicative senescence.

## Methods

### Cell culture, fluorescence-activated cell sorting (FACS) analysis, and senescence-associated β-galactosidase (SA-β-gal) activity

Early-passage (Young, ~25-27 population doublings[pdl]), late-passage (Senescent, ~60 pdl) human diploid 2BS fibroblasts (National Institute of Biological Products, Beijing, China), and HeLa cells were cultured in Dulbecco’s modified Eagle’s medium (Invitrogen) supplemented with 10% fetal bovine serum, 100 units/ml penicillin, and 100 μg/ml streptomycin, at 37 C in 5% CO2. Fluorescence-activated cell sorting (FACS) analysis, and senescence-associated β-galactosidase (SA-β-gal) activity were performed as described previously [5].

### Constructs and transfection

For the construction of vectors expressing flag-HuR, full-length coding region of HuR was amplified by PCR using flag-tagged primer GGAATTCATGGACTACAAGGACGACGATGACAAGTCTAATGGTTATGAA and primer GCTCTAGATTATTTGTGGGACTTGTTGG and inserted between EcoRI sites of pcDNA 3.1 vector (Clontech). The flag-HuR bearing an arginine-to-lysine mutation on residue 217 were generated using QuikChange® II Site-Directed Mutagenesis Kit (Stratagene) and inserted between EcoR and Xbal sites of pcDNA 3.1 (Clontech). For construction of vectors expressing CARM1 or control shRNAs, oligonucleotides corresponding to siRNA targeting CARM1 (CAGCTCT ACATGGAGCAGT), HuR (AAGAG GCAAUUACCAGUUUCA), or a control shRNA (AAGTGTAGTAGATC ACCAGGC) were inserted between the hind III and BgIIIsites in pSuper.retro (Oligoengine) vector following the manufacturer’s instructions.

All plasmid or siRNA transfection in HeLa cells were performed using lipofectamine 2000 (for plasmids) or oligofectamine (for siRNAs) (Invitrogen) following the manufacturer’s instructions. Cells were collected 48 h after transfection for further analysis. To establish lines stably expressing flag-HuR, flag-HuRΔ, or CARM1 shRNA, early-passage (~25 pdl) 2BS cells were transfected with a vector expressing flag-HuR, flag-HuRΔ, CARM1 shRNA, or with the respective control vectors by lipofectamine 2000 (Invitrogen) following the manufacturer’s instructions, selected by G418 (300 μg/ml, Invitrogen) for 3–4 weeks, and maintained in medium supplemented with 50 μg/ml G418.

### Preparation of cell fractions, immunoprecipitation (IP) assays, and Western blot analysis

Whole-cell lysates as well as cytoplamic and nuclear extracts were prepared as described previously [9]. IP assays were performed using 50 μg of whole-cell lysates and 1 μg of HuR or flag antibody (M2). For Western blot analysis, lysates were size fractionated by SDS-PAGE and transferred onto poly-vinylidene difluoride (PVDF) membranes. Monoclonal antibodies recognizing HuR, HDAC1, p16, c-fos, SIRT1, β-tubulin, and GAPDH were from Santa Cruz Biotechnologies (Santa Cruz, Calif.). Mouse monoclonal flag antibody (M2) was from Sigma. Mouse monoclonal mono/dimethyl arginine antibody (M/DMA) was from Abcam. After secondary antibody incubation, signals were detected by Super Signal West Pico Chemiluminescent Substrate (Pierce) following the manufacturer’s instruction.

### RNA-protein interaction assays and UV crosslink RNP IP assays

cDNA was used as a template for PCR amplification to generate the 3′UTR of different HuR target transcripts. All 5′ primers contained the T7 promoter sequence CCAAGCTTCTAATACGACTCACTATAGGGAGA-. To prepare the 3′-UTR fragments for cyclin A, cyclin B1, c-fos, SIRT1, and p16 mRNAs, primers (T7) CCAGAGACATAAATCTGTAAC and GGTAACAAATTTCTGGTTTATTTC for cyclin A 3′UTR, primers (T7) CTTGTAAACTTGAGTTGGAGT and TTTTTTTTTTTTGTATTTGAG for cyclin B1 3′UTR, primers (T7) GCAATGAGC CTTCCTCTGAC and CATTCAACTTAAATGCTTTTATTG for c-fos 3′UTR, primers (T7) AACTATCCATCAAACAAA and TATCCAGTCATTAAACAGT for SIRT1 3′UTR, and primers TTGGTCCCTCTTGATTAT and GTGATGTCTGGCTGTTTC for p16 3′UTR were used, respectively. For biotin pull-down assays, PCR-amplified DNA was used as template to transcribe biotinylated RNA by using T7 RNA polymerase in the presence of biotin-UTP, as described [9]. One microgram of purified biotinylated transcripts were incubated with 100 μg of cytoplasmic extracts for 30 min at room temperature. Complexes were isolated with paramagnetic streptavidin-conjugated Dynabeads (Dynal, Oslo), and the pull-down material was analyzed by Western blotting.

For cross-linking of RNP IP complexes, cells were exposed to UVC (400 mJ/cm^2^) and whole-cell lysates prepared for immunoprecipitation using monoclonal anti-HuR and polyclonal anti-AUF1 antibodies, as described [7]. The transcripts present in the RNP complexes were analyzed by real-time qPCR.

### RNA isolation, real-time qPCR, and mRNA half-life measurement

Total cellular RNA was prepared using RNeasy Mini Kit (Qiagen) following the manufacturer’s protocol. For real-time qPCR against GAPDH analysis to detect cyclin A, cyclin B1, c-fos, SIRT1, p16, and β-actin transcripts, primers TTGGTCCCTCTTGATTAT and GTGATGTCTGGCTGTTTC for cyclin A, primers GCACTTTCCTCCTTCTCA and CGATGTGGCATACTTGTT for cyclin B1, primers CGAAGGGAAAGGAATAAGATG and TGAGCTGCCAGGATGAACT for c-fos, primers TAGGCGGCTTGATGGTAATC and TCATCCTCCATGGGTTCTTC for SIRT1, primers GAAGGTCCCTCAGACATCCCC and CCCTGTAGGACCTTCGGTGAC for p16, and primers GTGGACATCCGCAAAGAC and AAAGGGTGTAACGCAACTAA for β-actin were used, respectively.

To measure the half-life of endogenous mRNAs, the expression of mRNA was shut off by adding actinomycin D (2 μg/ml) into the cell culture medium, whole cellular RNA was prepared at times indicated and subjected to real-time qPCR. Data were plotted as the mean ± SD from 3 independent experiments and the half-lives were calculated as previously described [7].

## Competing interests

The authors declare that they have no competing interests.

## Authors’ contributions

XZ and WW designed the study. LP, HT, NC, JY, LX, and BJ performed the experiments. MG, XZ and WW wrote the paper. All authors read and approved the final manuscript.
